# Emergencies in the COVID-19 Era: Less Attendances, More Admissions

**DOI:** 10.7759/cureus.25971

**Published:** 2022-06-15

**Authors:** Barbara Fyntanidou, George Stavrou, Aikaterini Apostolopoulou, Sofia Gkarmiri, Katerina Kotzampassi

**Affiliations:** 1 Emergency Department, AHEPA Hospital, Thessaloniki, GRC; 2 Leeds Institute of Emergency General Surgery, Leeds Teaching Hospitals NHS Trust, Leeds, GBR; 3 Department of Surgery, Aristotle University of Thessaloniki, Thessaloniki, GRC

**Keywords:** emergency department visits, hospital admissions, emergency department attendances, emergency, covid-19

## Abstract

Introduction

Healthcare systems suffered a significant hit by the COVID-19 pandemic since the spring of 2020, and a need for major reorganization emerged. Along with the constant increase in COVID-19 cases, a significant drop in emergency attendances for non-COVID-19-related conditions was noted worldwide. We decided to document attendances in our hospital's emergency department during the first lockdown period in order to monitor this trend, compare it to data from other countries, and start monitoring the effects of this reduction in the years to come.

Materials and methods

Emergency department attendances at AHEPA University Hospital, Thessaloniki, Greece, from March 10, 2020, to May 31, 2020, were documented and compared to the corresponding period in 2019. The data collected included the number of patients per specialty, severity upon admission, as well as the need for admission.

Results

We found a 58% reduction in emergency department attendance during the studied period compared to the corresponding period in 2019 (p<0.0001). The reduction was more noticeable in ears, nose, throat (ENT), and ophthalmology attendances (75.7% and 78.1% reductions, respectively, p<0.001), but other specialties, such as cardiology and general surgery, were also significantly affected (60% and 63% reductions, respectively, p<0.001). However, the percentage of attendances that required admission increased significantly by 25-33% (p<0.001) during the lockdown, reflecting the higher severity of cases reaching the hospital.

Conclusion

Despite the obvious reduction in attendances during the COVID-19 pandemic, patients still suffer from serious conditions that require hospital admission. Therefore, hospitals need to be supported to also care for these patients. The long-term effects of avoiding hospital attendance need to be closely monitored.

## Introduction

The outbreak of the SARS-CoV-2 virus was declared a pandemic on March 11, 2020, by the World Health Organization (WHO) [1]. Health systems had to prepare for the worst-case scenario, and most countries enforced strict lockdown measures to contain the spread of the virus. Various hospital departments, especially intensive care units (ICUs) and emergency departments (EDs), had to be thoroughly reorganized to cope with the new and unprecedented circumstances.

The Greek government applied a strict lockdown from March 13, 2020, to May 31, 2020 [2], and several major tertiary hospitals were appointed as COVID-19 reference centers. AHEPA University Hospital in Thessaloniki was one of those reference centers. Its emergency department had to be completely reorganized in order to manage a new and constantly evolving situation. Changes regarding the role of the personnel, the necessary skills and equipment, as well as the physical space and facilities, had to be implemented. AHEPA is one of the largest hospitals in Greece and a major tertiary referral center for the entire Northern Greece, managing all kinds of emergencies, in a 1:4 rota, with the exception of obstetric, gynecological, orthopedic, and urologic emergencies.

With the exception of confirmed or suspected COVID-19 cases, the number of patients with other urgent health issues attending the ED seemed to be surprisingly decreased during the period of the first lockdown. Similar statements have been made by many researchers worldwide [3-7]. Therefore, we decided to document the trend of ED attendances per specialty in our hospital during the first lockdown period and compare them to the corresponding period of the previous year. By documenting these trends and comparing them to findings from other countries, we aim to identify a pattern and also attempt to assess the effects of these reduced hospital attendances on the health status of the general population in the years to come.

## Materials and methods

This is a retrospective, observational study regarding the period of the first Greek lockdown. We searched the electronic database of the AHEPA University Hospital ED from March 10, 2020m to May 31, 2020, and from March 10, 2019, to May 31, 2019. The data that were collected include the number of incoming patients seeking medical help for non-COVID-19 related conditions, their severity scale upon presentation to ED (Emergency Severity Index 2019 - ESI), as well as the number of hospital admissions. 

We divided the study period into three smaller periods, corresponding to the different phases of the lockdown. The first period (March 10 - March 22, 2020, 13 days) was characterized by the suspension of educational (schools, colleges, universities) and sports activities and the gradual enforcement of hospitality and commercial business closure regulations. The second period (March 23 - May 4, 2020, 43 days) was the strictest lockdown period, where citizens were only allowed to move outside their houses for specific reasons. During the third period (May 5 - May 31, 2020, 27 days), businesses were gradually allowed to reopen, and normal, everyday activities were allowed to resume, with the benchmark of the schools reopening on June 1, 2020 [2]. 

Since this was an observational retrospective study with no identifiable patient information, the ethics committee approval was not required.

Statistical analysis

One-way analysis of variance (ANOVA) was used for between and within-group comparisons, followed by Tukey-Kramer and LSD (post hoc tests). Data are presented as mean±SD. For statistical analysis purposes, Prism software version 9.2.0 for Windows was used (GraphPad Software, Inc., CA, USA), while a p-value of less than 0.05 was considered statistically significant.

## Results

The total number of patients attending the ED during the three study periods in 2020 was 3,304, compared to 10,172 during the corresponding time frame in 2019 (58% reduction, p<0.0001). By further analysis of each studied timeframe, we documented 508, 1378, and 1418 ED attendances for each study period in 2020 vs. 1580, 5183, and 3409 ED attendances for the respective study periods in 2019. This corresponds to a decrease in ED attendances by 67.8% (p=0.0005), 73.4% (p<0.0001) and 58.4% (p<0.0001), respectively. 

However, the number of days of each study period is not similar to the rest. Therefore, we calculated the mean ED attendances per day for each study period. For each study period in 2020, we documented 39, 32, and 38.8 ED attendances per day, vs. almost a triple numbers of 121.5, 120, and 92.1 ED attendances per day for the corresponding study periods in 2019 (p<0.0001).

A more detailed description of the distribution of ED attendances among specialties is shown in Tables 1-3.

**Table 1:** ED attendances and admissions/visit during the first lockdown period (13 days) compared to 2019 NS - not significant

Specialty	2020			2019			
	Visits	Admissions	Admissions/visit %	Visits	Admissions	Admissions/visit %	p
Internal medicine	208	91	43.75	544	220	40.4	NS
Neurology	49	24	49	141	62	43.9	NS
Cardiology	71	39	54.9	208	101	48.5	NS
General surgery	53	26	49.1	193	62	32.1	0.0343
ENT (ear, nose, throat*)*	71	5	7.1	227	17	7.5	NS
Ophthalmology	44	8	18.2	229	13	5.7	0.011
Psychiatry	12	7	58.3	38	14	36.9	NS
Total	508	200	39.4	1580	489	31	0.0005
Patients/day	39	15.4		121.5	37.6		

**Table 2:** ED attendances and admissions/visit during the second lockdown period (43 days) compared to 2019 NS - not significant

Specialty	2020			2019			
	Visits	Admissions	Admissions/visit %	Visits	Admissions	Admissions/visit %	p
Internal medicine	519	267	51.4	1642	681	41.5	<0.0001
Neurology	131	94	71.8	443	219	49.4	<0.0001
Cardiology	205	137	66.8	649	328	50.5	<0.0001
General surgery	155	60	38.7	591	189	32	NS
ENT (ear, nose, throat)	165	32	19.4	962	67	7	<0,0001
Ophthalmology	151	18	11.9	818	35	4.3	0.0003
Psychiatry	52	21	40.4	78	23	29.5	NS
Total	1378	629	45.6	5183	1542	29.7	<0.0001
Patients/day	32	14.6		120	35.9		

**Table 3:** ED attendances and admissions/visit during the third lockdown period (37 days) compared to 2019 NS - not significant

Specialty	2020			2019			
	Visits	Admissions	Admissions/visit %	Visits	Admissions	Admissions/visit %	P
Internal medicine	291	200	68.7	1055	371	35.2	<0,0001
Neurology	159	105	66.1	299	129	43.1	<0,0001
Cardiology	222	134	60.4	389	191	49.1	0.0094
General surgery	237	105	44.3	419	116	27.7	<0.0001
ENT (ear, nose, throat)	204	25	12.2	618	44	7.1	0.0317
Ophthalmology	257	16	6.2	553	24	4.3	NS
Psychiatry	48	29	60.4	76	24	31.6	0.0029
Total	1418	614	43.3	3409	899	26.4	<0.0001
Patients/day	38.3	16.6		92.1	24.3		

The greatest reduction in attendances was observed for the ear, nose, throat (ENT), and ophthalmology specialties (75.7% and 78.1% reduction, respectively, p<0.001) throughout the lockdown period.

Conversely, there is a distinctively smaller reduction in the number of psychiatric ED attendances (33.45%) during the second period of the complete lockdown.

The number of admissions across the three study periods of 2020 was found to be 1443 (200, 629, and 614 for each corresponding period), vs. a total number of 2930 in 2019 (489, 1542, and 899, respectively), demonstrating a reduction of 51.8% (p<0.001).

However, if we correlate the number of attendances with the number of admissions at any study period, we observe a significantly increased rate of admissions during the lockdown. More specifically, for the three study periods in 2020, 39.4%, 45.6%, and 43.3% of the ED attendances needed to be admitted. The corresponding data for the same periods in 2019 were 30.9%, 29.7%, and 26.4% (p<0.001), which demonstrates an increase of 25-33% in the rate of admissions during the 2020 lockdown.

Furthermore, the number of admissions to the department of internal medicine was almost doubled (68.7% in 2020 vs. 35.1% in 2019, 95.7% increase, p<0.001) during the third study period, as well as the number of admissions to the psychiatric wards (60.4% in 2020 vs. 31.6% in 2019, 91.1% increase, p<0.001). Similarly, we identified a notable increase in regarding the admissions to the department of general surgery during the same time period (44.3% in 2020 vs. 27.7% in 2019, 60% increase, p< 0.001), whereas a smaller but significant increase was noted during the second and third lockdown period regarding the admissions to the department of neurology (increase by 45% and 53.1%, respectively, p<0.001) and those to the department of cardiology (32.3% and 22.8% respectively, p<0.01).

## Discussion

The effect of the COVID-19 outbreak on healthcare systems worldwide was unprecedented. Hospitals had to be reorganized, staff retrained, and redeployed to meet the demands of the pandemic. Along with a significant reduction or even a complete cessation of elective surgery, in order to preserve resources for the critically ill COVID-19 patients [8], a significant reduction in the number of patients seeking medical help for non-COVID-19 conditions was noted, an effect noticed across multiple countries [9-13]. Recently, the term "COVID collateral damage syndrome" has been utilized to describe the effect that delayed or unavailable medical care may have on patients that require intensive or urgent care [14].

Possible explanations for the above could be the profound fear of a new, unknown and deadly virus that lingered in the community, forcing people to stay indoors and avoid attending hospitals for fear of being infected. This may have played a significant role in our hospital, as AHEPA University Hospital was the only designated COVID-19 reference tertiary hospital in the city of Thessaloniki at the time, with a population of approximately one million people. Another reason, especially in countries with well-organized primary health care systems, could be the thorough gatekeeping by general practitioners that prevented non-urgent cases from reaching the hospitals [15].

This was also noticed in our hospital, with a total reduction of 58% in ED attendances (3304 vs. 10,172 in 2019, p<0.0001), matching, if not surpassing, similar reductions in Germany (35%), France (41.47%), USA (42%) and Italy (66.2%) [10,12,13,16]. This trend shows some signs of reversal during the second and, especially, the third phase of the lockdown; however, it never reached the level of attendances noted in 2019.

By further analyzing the attendances per different specialties, the most significant reduction was noted in ENT and ophthalmology emergencies, which were reduced by 75.7% and 78.1%, respectively. This matches the findings from other countries [12], where ENT, ophthalmology, and dermatology attendances were among the first to be diminished. A possible reason for that could be that emergencies of these specialties could be managed at home with over-the-counter (OTC) medication before attending the ED of a hospital.

An interesting finding of our study was that the attendances for even the "emergency front-line" specialties, such as cardiology and general surgery, were also significantly reduced compared to 2019 (63% for general surgery and 60% for cardiology, p<0.001). Such a prominent reduction in emergency surgical cases could be partially attributed to reduced incidence of trauma, as most people were staying at home; thus, there were fewer road traffic accidents and less trauma associated with daily activities. The reduction in emergency cardiological cases has also been described elsewhere, with myocardial infarctions and strokes being significantly decreased [10,12,17-19]. However, it is assumed that a number of deaths possibly due to myocardial infarction was undocumented as such because they remained undiagnosed due to the reluctance of the patients to visit the hospital [20].

Another possible explanation for this could be that people spent more time at home, even working from home, thus avoiding more stressful work environments that can trigger cardiovascular incidences. Furthermore, it is possible that more people undertook aerobic exercise such as walking or jogging as a means to get out of home, which, in turn, had a beneficial effect on the incidence of acute cardiovascular events [15].

Another interesting finding was the fact that at the first phase of lockdown, psychiatric ED attendances were also reduced similarly to other specialty attendances, albeit not significantly. This reduction, however, began to reverse during the second and, mainly, the third period of the lockdown, and this, combined with the increased number of admissions per attendance during the third period (60.4% in 2020 vs. 31.6% in 2019, p<0.001), clearly reflects the psychological burden that the strict lockdown inflicted upon the Greek population, along with the associated insecurity about the future and the socioeconomic consequences. This is also in accordance with findings from other countries, such as Iran and USA [16,21,22].

However, it should be emphasized that, despite the obvious reduction in attendances, the number of patients that required hospital admission for non-COVID-19 related conditions seems significantly increase throughout the lockdown. A possible reason could be that patients, most likely for fear of contracting COVID-19, avoided attending hospitals until their condition did not allow them to defer it any longer. This match reports from other hospitals, especially in relation to emergency surgical cases [23,24] or the need for higher-level transfers [16].

All of the above clearly demonstrates the reduction in the number of ED attendances during the first wave of the COVID-19 pandemic and the first and strictest lockdown. This reduction, however, does not come without a cost, given the increased percentage of admissions per ED visit, which reflects the higher severity of cases reaching the hospital. Furthermore, the potential effect of this reduction on long-term outcomes, combined with the temporary cessation of elective activities in order to preserve resources, is, as of yet, unknown and needs to be closely monitored.

A potential limitation to our study is that it is a single-center study and no other data are available regarding ED attendances in other hospitals in Thessaloniki. Another limitation is that AHEPA University Hospital was the only COVID-19 reference tertiary hospital at the time, which may have played a part in the public avoiding it for fear of infection. However, it should also be noted that it is located right in the city center and within the university campus, making it very easily accessible and attracting the greatest number of ED attendances and, therefore, potential admissions.

## Conclusions

Dealing with this pandemic, patients and health professionals had to overcome many boundaries. However, preserving a high level of healthcare quality is of the utmost importance. Despite the dynamic of the pandemic, COVID-19 is not the only condition that afflicts our patients, and the same pathological backgrounds and conditions that require urgent care are still present in the community. Clear messages about the necessity of still attending hospitals for serious symptoms should be given to citizens, and they should be encouraged to do so, while health systems should be reinforced with facilities and personnel in order to meet the patients' needs safely and effectively. The effect that the pandemic, the lockdowns, and especially the cessation of elective activities will have on the general public health in the next five to ten years is an interesting area of research, and the data collection has already begun.
